# Acquisition of endoscopic nasal surgery skills with a lamb’s head model

**DOI:** 10.1016/j.bjorl.2022.05.009

**Published:** 2022-06-09

**Authors:** Henrique F. de Oliveira, Valdes Roberto Bollela, Wilma Terezinha Anselmo-Lima, Donald Matthew Pianto, Carlos Augusto C.P. de Oliveira, Márcio Nakanishi

**Affiliations:** a Universidade de Brasília (UnB), Faculdade de Medicina, Programa de Pós-Graduação em Ciências Médicas, Brasília, DF, Brazil; b Universidade de São Paulo (USP), Faculdade de Medicina de Ribeirão Preto (FMRP), Departamento de Prática Médica, Ribeirão Preto, SP, Brazil; c Foundation for Advancement of International Medical Education and Research, Philadelphia, United States; d Universidade de São Paulo (USP), Faculdade de Medicina de Ribeirão Preto (FMRP), Departamento de Oftalmologia, Otorrinolaringologia e Cirurgia de Cabeça e Pescoço, Ribeirão Preto, SP, Brazil; e Universidade de Brasília (UnB), Programa de Pós-Graduação em Computação Aplicada, Departamento de Ciência da Computação, Brasília, DF, Brazil; f Universidade de Brasília (UnB), Faculdade de Medicina, Departamento de Otorrinolaringologia, Brasília, DF, Brazil

**Keywords:** Skills acquisition, Endoscopic surgery, Lamb model, Patient safety, Surgical training

## Abstract

**Objectives:**

To evaluate the acquisition of surgical skills by otolaryngology residents and established the minimum number of dissections of a lamb’s model to be performed before practicing on human patients.

**Methods:**

Nineteen second-year otolaryngology residents performed ten dissections each, five on each nasal cavity, always practicing the same three surgical procedures on the lamb model. Each student’s training lasted 2-months, and the entire training intervention lasted 4-years, over four generations of residents. All dissections were recorded and were selected at random for examination by two independent otolaryngology surgeons, who were otherwise not involved in the research. Assessment of the 190 dissections used an instrument validated for surgical training of medical residents.

**Results:**

To a 1% significance level, statistical analysis revealed increased performance and satisfactory results were observed after the sixth dissection. Furthermore, after the eighth dissection, skill acquisition was relevant and sustained.

**Conclusion:**

Training in endoscopic nasal surgery on a lamb’s head model improves surgical skills and handling of surgical instruments. Our results showed the relevance of the lamb model for training in otolaryngology surgery, impacting on patient safety.

**Level of evidence:**

2.

## Highlights

• After eight repetitions, there is a statistically significant increase in total score.
• The mean duration of dissections decreased from 33 to 15 min during training.
• The lamb is a useful tool in the dissection learning process.

## Introduction

Nasal endoscopic surgery has become the preferred method for the majority of rhinology procedures. However, these techniques are challenging, both to learn and to teach. The fact that surgery is performed in small, narrow spaces and the surgical site is surrounded by structures including large vessels and nerves generates dilemmas when performing and teaching nasal endoscopy.^1^, ^2^

Although dissection of human cadavers is the ideal way to learn, it is becoming increasingly expensive and is made difficult by ethical and legal issues in many countries. These obstacles to using human heads have resulted in the development of alternatives over recent years.

In otorhinolaryngology, it is clear that in terms of anatomy, lamb’s heads offer many similarities to human nasal passages^3^, ^4^, ^5^ and so they have been considered as a tool for learning in many locations.^6^, ^7^, ^8^

Comparative anatomical studies were conducted and structures that are sufficiently similar in the lamb to those in the human for use in training were identified. The lamb model was then validated as an instrument for teaching nasal endoscopic surgery.^9^

Precise and extended training is an indispensable element of an otorhinolaryngology resident’s medical training, for reasons of ethics and patient safety.^10^ Acquisition of essential surgical skills before students start to practice on live patients reduces the time taken to achieve the level of competence needed for autonomous practice. Such practice is associated with better results, which are manifest in lower morbidity and reduced pharmacological and personnel costs.

It remains to be shown whether nasal dissection of the lamb model actually enables residents to acquire the skills essential for perfecting specialist otorhinolaryngology training.

The objective of this study was to evaluate acquisition of endoscopic nasal surgery skills by otolaryngology residents during training with a lamb’s model and establish the minimum number of dissections to be performed before practicing on human patients.

## Methods

This study was initiated after full authorization by the Animal Research Ethics Commission of our institution. All of the residents involved were informed about the study and agreed to take part in advance through an informed consent form. All dissections conducted by residents were supervised by the same instructor and were video recorded, from 2013 to 2016.

The lamb’s head was placed in a support for handling as a model, as described by De Oliveira et al.^11^ The instrument employed was a HOPKINS® Karl Storz 0° rigid scope, 4-mm in diameter and 18-cm in length. This was fitted with an Optice 2.0 USB camera (Doctus Equipamentos Médicos), connected to a laptop computer. An LED portable light source was also used. Digital images of the procedures were viewed and stored for later analysis.

Before performing their first dissection, each of the residents watched a demonstration video showing a standard dissection conducted by two specialists (researchers) which made clear the steps involved and the surgical objectives to be practiced.

The researchers chose three operations (inferior nasal concha surgery; ethmoid bullectomy; and maxillary antrostomy) for the residents to perform during training. These procedures were chosen because they are commonly performed on humans and are totally reproducible in the animal model, due to the anatomic similarity.

Inferior concha surgery was performed with the scope in the left hand, using Heyman scissors (Karl Storz Heymann Nasal Scissors). The surgical method chosen was linear partial inferior turbinectomy: head, body, and tail. Ethmoid bulla surgery was performed after medial luxation of the medial concha, after which Grunwald forceps (Karl Storz Grunwald-Henke nasal cutting forceps) were used to remove the entire anterior portion of the bulla. Maxillary antrostomy was performed after removal of the uncinate process with a sickle knife (Karl Storzsickle knife) and the procedure was considered complete when the entire maxillary fontanelle had been exposed.

All dissections were recorded. Each resident performed the dissections ten times, five in each nasal fossa, using a total of five lamb’s heads, on alternate sides, starting on the left. The time elapsed between each resident’s first and last dissections was 6–8-weeks. All training was done before performing the respective surgeries on patients.

All of the residents were in the second semester of their second year on the medical residency program. In other words, each year only second-year residents dissected the lambs.

After all dissections had been performed, the videos were examined independently by two otorhinolaryngologists who were not the researchers and were experienced nasal surgeons. Videos were randomized so that examiners did not know which resident was conducting each procedure or the chronological order of dissections. Assessments were therefore blind, and the sequence of the videos was randomized.

Dissections were assessed using a validated tool for surgery training of residents called the Objective Structured Assessment of Technical Skill (OSATS).^12^

After analyzing each video, each examiner awarded a score (1, 3, or 5) to each of the five items analyzed (Table 1). Each video was therefore given a total score in the range of 5–25 and each examiner awarded ten scores for each resident’s dissections (Table 2).

**Table 1:** Items assessed in each dissection.

**Respect for tissue**	Frequently used unnecessary force on tissue or caused damage by inappropriate use of instruments	Careful handling of tissue but occasionally caused inadvertent damage	Consistently handled tissues appropriately with minimal damage
	1	3	5
**Time and motion**	Many unnecessary moves	Efficient time/motion but some unnecessary moves	Economy of movement and maximum efficiency
	1	3	5
**Instrument handling**	Repeatedly makes tentative or awkward moves with instruments	Competent use of instruments although occasionally appeared stiff or awkward	Fluid moves with instruments and no awkwardness
	1	3	5
**Flow of operation and forward planning**	Frequently stopped operating or needed to discuss next move	Demonstrated ability for forward planning with steady progression of operative procedure	Obviously planned course of operation with effortless flow from one move to the next
	1	3	5
**Knowledge of specific procedure**	Deficient knowledge. Needed specific instruction at most operative steps	Knew all important aspects of the operation	Demonstrated familiarity with all aspects of the operation
	1	3	5

**Table 2:** Examiners’ scores.

Resident	Examiner 1										Examiner 2									
	Dissection										Dissection									
	1	2	3	4	5	6	7	8	9	10	1	2	3	4	5	6	7	8	9	10
1	5	11	15	21	19	21	25	21	21	23	11	21	25	15	15	25	23	23	21	23
2	9	15	9	17	17	9	19	17	21	21	19	19	21	11	19	19	17	25	21	25
3	5	7	9	5	5	5	9	11	17	19	9	9	7	11	13	11	21	17	19	19
4	9	5	5	11	17	15	5	11	15	23	11	11	13	15	21	19	11	21	11	25
5	5	5	5	5	7	5	5	5	5	11	9	15	7	15	11	15	5	9	11	19
6	9	9	5	11	9	5	5	5	19	9	9	13	15	7	15	11	13	5	13	13
7	5	5	9	5	15	11	17	13	17	15	5	15	13	11	13	21	11	9	21	15
8	5	11	11	7	5	13	21	5	13	15	13	17	15	15	17	23	19	23	23	23
9	5	11	21	17	13	5	13	9	21	17	13	13	15	13	21	21	9	23	17	25
10	5	5	15	21	11	9	15	7	11	17	13	17	21	23	11	17	21	13	19	9
11	13	7	25	21	17	11	11	15	19	23	15	17	25	19	25	17	17	17	19	21
12	5	17	5	5	11	5	15	5	15	15	11	15	11	13	15	19	21	13	11	21
13	7	15	25	13	17	17	9	19	21	21	15	15	23	17	17	19	15	13	21	23
14	5	7	9	5	5	5	9	7	21	21	9	13	7	11	15	7	21	17	23	21
15	5	5	17	5	9	5	5	9	15	15	5	7	19	19	21	11	17	15	21	25
16	5	9	9	15	11	5	5	9	15	7	5	15	13	17	19	9	17	11	11	19
17	11	15	9	5	5	13	5	7	13	15	9	15	11	13	13	11	21	13	15	15
18	5	7	15	7	7	5	15	17	19	19	11	9	15	11	9	13	25	21	21	21
19	11	5	11	19	5	13	7	13	15	17	7	11	21	15	17	19	7	13	17	17

These data were then analyzed statistically. First, the median for all of the residents was calculated for the total scores for each dissection. The Wilcoxon test was then applied. This is a nonparametric test that does not require that data be distributed in ascending, linear order, which is ideal for our study, since performance in each dissection could vary without any underlying pattern.

## Results

A total of 19 residents (six men and thirteen women) performed all three operations in ten dissections and 190 videos were recorded. Each video was scored from 5 to 25 by each examiner, blinded and independently. Videos were analyzed in random order and then the scores were tabulated in chronological order by the same person who had randomized the dissections.

**Figure 1:**
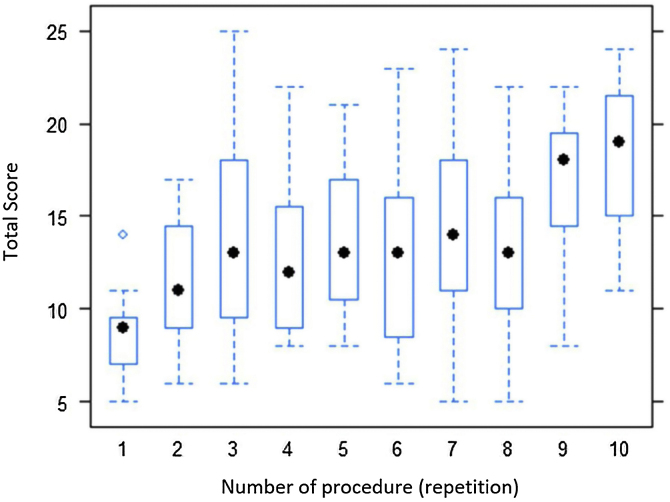
Median score for each series of dissections.

The graph (Fig. 1) shows the distribution of scores for each series of procedures. The three different procedures performed in each dissection were analyzed in conjunction.

With regard to Fig. 1: the dot in the center of each box indicates the median score for that repetition for all 19 residents. It can be observed that there is a large increase in scores up to the third repetition, followed by an oscillation until the eighth repetition, and then a considerable increase up to the tenth.

The initial step in statistical analysis was to test the median score for each of the ten repetitions against a score of 15 (representing adequate performance: mean score of three for each of the five components analyzed). We therefore tested whether total median score for each repetition was equal to 15 or less than 15 (*H*_0_: *m* = 15 vs. *H*_1_: *m* < 15). Rejection of the null hypothesis indicates median performance below 15.

The results in Table 3 show descriptive values (*p*-values) for the test *H*_0_: *m* = 15 versus *H*_1_: *m* < 15. For 5% significance, *p*-values less than 0.05 indicate rejection of the null hypothesis. We can observe that, for the first six repetitions, the null hypothesis of adequate performance is rejected in all cases, except the third repetition. Thereafter, from the seventh to the tenth repetitions, the null hypothesis of adequate performance cannot be rejected.

**Table 3:** *p*-Values for the test of *H*_0_: *m* = 15.

1	2	3	4	5	6	7	8	9	10
0.0001	0.0006	0.1539	0.0244	0.0480	0.0289	0.1654	0.0648	0.9877	0.9988

We then conducted multiple comparisons analysis, comparing the median for each repetition (rows in the table) with the medians for later repetitions (columns). Thus, the median first repetition score (i = 1) was compared with following nine repetitions (j = 2 to j = 9); the median second repetition score (i = 2) was compared with the following eight repetitions (j = 2 to j = 10), and so on. We therefore tested the null hypothesis *H*_0_: *m_i_* = *m_j_* versus *H*_1_: *m_i_ <* *m_j_* for *i < j*, where *i* and *j* represent the number of repetitions (Table 4). The results shown are *p*-values.

**Table 4:** *p*-Values for the test of *H*_0_: *m_i_* = m_j_.

	j = 2	j = 3	j = 4	j = 5	j = 6	j = 7	j = 8	j = 9	j = 10
**i = 1**	0.0009	0.0006	0.0004	0.0004	0.0005	0.0009	0.0005	0.0004	0.0004
**i = 2**	–	0.0507	0.0868	0.0218	0.0893	0.0179	0.0431	0.0004	0.0004
**i = 3**	–	–	0.2977	0.2496	0.2833	0.1940	0.2496	0.0044	0.0014
**i = 4**	–	–	–	0.0431	0.1940	0.0893	0.1392	0.0024	0.0011
**i = 5**	–	–	–	–	0.2701	0.1322	0.2496	0.0019	0.0004
**i = 6**	–	–	–	–	–	0.0893	0.1461	0.0015	0.0004
**i = 7**	–	–	–	–	–	–	0.2496	0.0029	0.0011
**i = 8**	–	–	–	–	–	–	–	0.0005	0.0004
**i = 9**	–	–	–	–	–	–	–	–	0.3187

In this analysis, we set the significance level at 1%, to be more rigorous in identifying improved scores and, consequently, performance. Observing the first line of Table 4, there was an increase in median total score after the first time the procedure was performed.

In all of the rows from 2 to 8, equality of medians from the second to eighth repetition of the procedure cannot be rejected, but the median does increase from the ninth to the tenth repetition. Thus, after eight repetitions, there is a statistically significant increase in total score. The ninth row shows that there was no evidence of improvement between the ninth and tenth repetitions.

The mean duration of dissection was 33 min for the first series, whereas the last series took 15 min. Over the entire series of 190 dissections, the mean time taken by the residents to perform the three operations was 23 min.

## Discussion

There was clear evidence of acquisition of nasal endoscopic surgery skills during training with the lamb model. In general, performance improved as the residents repeated the procedures chosen, especially over the first three and last two dissections, and there was an enormous improvement comparing the ninth and tenth dissections with the first (Fig. 1).

There is an oscillation in mean results from the third to eighth repetition, with rises and falls in performance, which is to be expected in a learning process. One interesting observation is that a pattern can be identified despite this variation. The falls in mean scores all occurred on the even-numbered repetitions (4, 6, and 8), all dissections performed on the right nasal fossa, which was the side that several residents reported being the most difficult.

Another important detail that emerges from statistical analysis is that, from the eighth dissection onwards, there is a satisfactory and sustained increase in surgical skills. We can therefore conclude that before medical residents practice this surgery on live patients, they should practice with the lamb model a minimum of eight times.

If we make a simple comparison between the mean time taken for the last and first dissections, the final duration was less than half of the time taken for the first series. Delgado-Vargas et al.^13^ assessed four residents and observed a reduction in dissection time over the course of training.

**Figure 2:**
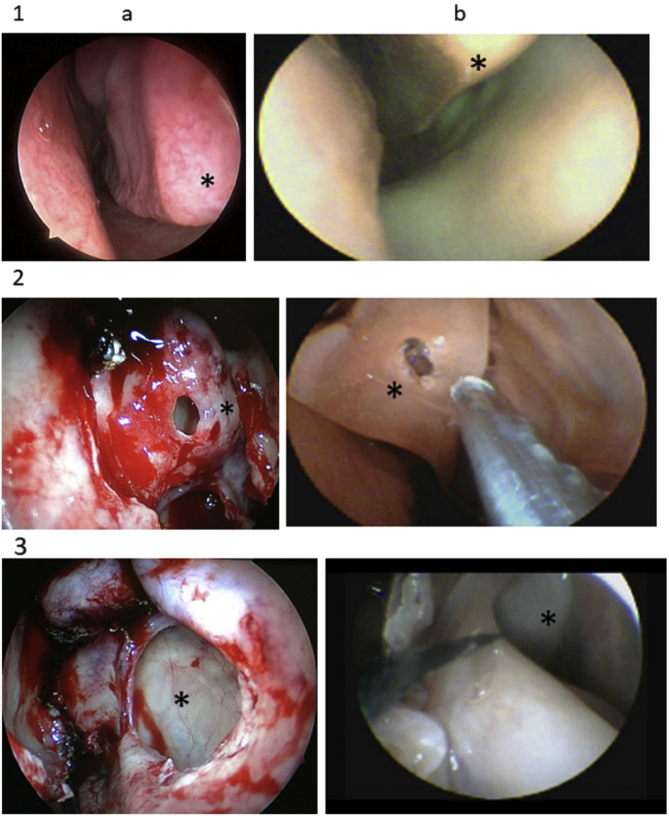
Human (a) and lamb (b) anatomy. 1. Inferior turbinate (*); 2. bullectomy (*); 3. maxillary sinus (*).

Animal model shave been adopted in response to difficulties related to cadavers and the limitations of other options. The lamb’s head has been widely studied as a model for nasal endoscopic surgery and its similarities to the human head have been described, enabling its use as an important teaching resource (Fig. 2). It is also inexpensive and easily reproduced.

The lamb’s head has been validated before as a teaching resource for nasal surgery. However, a single dissection was performed by different groups: medical students, residents at different stages, fellows, and experienced otorhinolaryngologists. Performance improved as the level of education and knowledge of each group increased.^9^

Our study is novel in terms of the systematic training conducted with this validated model. It comprises a guide for acquisition of basic surgical skills and adequate handling of instruments and includes a method for measuring progress. Other studies have already shown acquisition of skills using virtual simulators.^14^, ^15^ Fortes et al.^16^ used a real synthetic model and also found evidence of its utility for training to improve the surgical skills of otorhinolaryngologists.

We only analyzed residents in the second half of their second year of the residency program, in contrast with previous studies that have mixed students with differing levels of knowledge.^9^, ^17^ This meant the data were collected over a period of 4-years (four generations of residents) to recruit a sample comprising residents with the same level of knowledge. We therefore innovate by eliminating a source of selection bias, proving that the model enables skill acquisition.

The dissection training using an animal model proposed here is not intended to teach how to treat human diseases. However, once these surgical skills have been acquired through dissection of the model, it will be easier for students to begin practicing on humans, still under supervision. Zuckerman et al.^18^ have already demonstrated how practice with human cadavers enables surgeons to acquire skills.

Furthermore, recording the dissections enables analysis of performance and evaluation of the learning process, at any point. It also means that students can practice on their own, without a need for real-time supervision, since this can be provided later, by reviewing the videos.

The anatomic parameters of the three procedures conducted for this study are very similar to those observed in human patients, as are the textures of the tissues. The surgical steps were chosen on the basis of previous studies of comparative anatomy.^3^, ^4^ Essentially, the only significant difference is that the inferior concha is more elongated in the lamb.

It should be pointed out that the objective was not to compare one medical resident with another. Comparisons were always made between videos of the same student, analyzing their performance conducting the three procedures. This revealed the progress that each one made, respecting individual characteristics, and making it possible to measure acquisition of skills.

The overall objective of the entire study is patient safety. As training progresses, it enables surgeons to perfect and mature their technique. The lamb is a useful tool in this learning process, but also reveals a limitation since a human cadaver would be the ideal material.

## Conclusion

In addition to its similarity, the lamb’s head model proved adequate for acquisition of surgical skills (for the three procedures) after a minimum of eight repetitions, resulting in significant and sustained improvements. Use of this model in the basic stage of training of otorhinolaryngological surgeons is recommended since it will improve the quality of training which will undoubtedly result in benefits and greater safety for patients.

## Funding

This research received no specific grant from any funding agency in the public, commercial, or not-for-profit sectors.

## Data availability

All relevant data are within the paper.

## Conflicts of interest

The authors declare no conflicts of interest.
